# A Spatial Analysis of Food Insecurity and Body Mass Index with Income and Grocery Store Density in a Diverse Sample of Adolescents and Young Adults

**DOI:** 10.3390/nu15061435

**Published:** 2023-03-16

**Authors:** Joanna Buscemi, Alexander O’Donnell, Mary Takgbajouah, Paige Patano

**Affiliations:** Department of Psychology, DePaul University, Chicago, IL 60614, USA

**Keywords:** geographic information systems, food insecurity, body mass index, socioeconomic status, grocery store access

## Abstract

Food insecurity occurs when a household lacks consistent access to food and is more prevalent in ethnic and racial minority populations. While there has been a proliferation of research linking food insecurity to obesity, these findings are mixed. It may be helpful to consider some additional geographic factors that may be associated with both factors including socioeconomic status and grocery store density. The purpose of the current study aimed to examine spatial relationships between food insecurity and SES/store density and BMI and SES/store density in a diverse sample of adolescents and young adults across two studies in a large, urban city. GIS analysis revealed that participants with the highest food insecurity tend to live in the zip codes with the lowest median income. There did not appear to be clear a relationship between food insecurity and store density. Participants with the highest BMI tend to live in zip codes with lower median income and participants with higher BMI tended to live in the south and west sides of Chicago, which have a relatively lower concentration of grocery stores in the city. Our findings may help to inform future interventions and policy approaches to addressing both obesity and food insecurity in areas of higher prevalence.

## 1. Introduction

Food insecurity occurs when a household lacks consistent access to food. It affects more than 13.5 million households across the U.S., and close to 33.8 million individuals nationwide [1,2]. Rates of food insecurity have increasingly grown since 2005, but it does not affect all Americans equally [3]. Individuals with food insecurity are more likely to have completed less formal education, be female, come from racial or ethnic minority groups, live in a household with children, and have a lower income when compared to individuals without food insecurity [1,3,4]. In particular, Black individuals are almost 70% more likely to be food insecure in comparison to white individuals, while Hispanic individuals are 24% more likely to be food insecure than white individuals [3]. This is concerning, as food insecurity is associated with numerous negative health outcomes in both children and adults, including increased rates of depression, anxiety, anemia, metabolic and cardiovascular diseases, and obesity [1,4,5].

Obesity is defined as an excess of body fat that has the potential to lead to a number of adverse health outcomes, such as diabetes, heart disease, and several cancers, including breast, ovarian, prostate, and colon cancer [6]. Since the 1970s, global obesity rates have quadrupled, and obesity is currently the number two cause of early death in the United States and Europe [2,6]. In the U.S, more than a third of adults have obesity, and almost 20% of children have obesity [6,7].

A large body of research has linked obesity to food insecurity; it is posited that those with food insecurity may engage in patterns of consumption that can increase the storage of fat [2,4,5]. For example, individuals may restrict their diet due to a lack of access to food, and then as a result overeat when there is an availability of food [2,4,5]. This binge and restrict cycle negatively impacts metabolic functioning and promotes the storage of fat in the body [2]. Additionally, food insecurity may also increase consumption of low-cost foods that are high in calories but low in nutrients, which can also promote obesity [2,4,5,8,9].

While there is empirical support for the obesity/food insecurity relationship, some results are mixed. For example, some studies have found differential associations across minoritized racial and ethnic groups, while other studies have found no differences [2,5]. When looking at sex differences, some studies have found no significant effects of food insecurity on obesity in men or children while finding a significant effect in women [1,9], with one finding that this significant effect only occurred among women experiencing short term food insecurity [1]. Furthermore, another study found that the relationship between food insecurity and obesity has been shown to be stronger in females with lower incomes when compared to males with lower incomes, suggesting a differential susceptibility to obesity by sex in individuals with lower incomes [3]. Additionally, a commentary on gaps in research on the relationship between the above-mentioned constructs also found that the association between food insecurity and obesity is particularly strong in women, compared to men [4], while another study found that food insecurity was significantly linked to obesity among women [2]. Overall, previous research seems to suggest that food insecurity may be associated with obesity among women, but not men.

Given the equivocal findings regarding associations between obesity and food insecurity, it may be helpful to consider some additional geographic factors that may be associated with both factors, including socioeconomic status and grocery store density. Some research has shown that increased access to nutritious food options is associated with increased diet quality, and that living a shorter distance to the nearest supermarket is associated with better dietary behaviors and lower body mass index (BMI) [2,10,11]. On the other hand, research has also shown that grocery shopping at discount stores, grocery shopping at stores situated in low-income neighborhoods, and being Black or African American are associated with increased BMI and poorer diet quality [12]. Other research has found no significant results when investigating these relationships [12]. Geographic information systems (GIS) approaches to analyzing data can be helpful in mapping where inequities lie in terms of obesity and food insecurity and how these factors may cluster by region and SES/grocery store density. Identifying neighborhood-level inequities and patterns may help to inform future interventions and policy approaches to addressing both obesity and food insecurity in areas of higher prevalence. Previous GIS analyses have revealed significant associations between food swamps and deserts, income, and health outcomes, including obesity, but have not examined the role of self-reported food insecurity [13]. This specific factor may be important as individuals living in food oases may have limited access to food for a number of reasons, and vice versa.

The purpose of the current study aimed to examine spatial relationships between food insecurity and SES/store density and BMI and SES/store density in a diverse sample of adolescents and young adults in the Chicagoland area. Given findings from previous research, we also explored these relationships by race/ethnicity and sex. Given that minoritized racial and ethnic groups are typically under-represented in research [4], it is important to study these constructs in a diverse sample. GIS analysis may reveal concentrated areas of higher BMI and/or food insecurity and may reveal patterns of these concentrations by income/grocery store density. In addition to the GIS analysis, we conducted regression analyses to determine whether there were any statistically significant relationships between our variables of interest. Based on previous literature, we hypothesized that there would be a visible pattern of higher food insecurity in areas of poverty and lower store density. We also hypothesized that there would be a visible pattern of higher BMIs in areas of poverty and lower store density. We hypothesized that these patterns would be exacerbated for Black and Latinx participants and that food insecurity would be more pronounced in females than males. Finally, we hypothesized that these patterns would be confirmed with our inferential statistical analyses.

## 2. Materials and Methods

### 2.1. Participants

Data included in this study came from two separate studies [14,15]. We included data from both studies to increase our sample size and to be able to include participants from across the adolescent—early adult developmental period. The Study 1 sample was high school students across 4 schools and was a mixed methods study including quantitative and qualitative data. Study 2 was a community sample of young adults and utilized a cross-sectional, quantitative design. Both samples were diverse in terms of race/ethnicity and income and representative of the city and surrounding suburbs.

*Study 1*: The participants were high school students across 4 public schools in Chicago. Of those who responded (*n* = 47), 44.7% were female, 68.1% were Black, 6.4% were Latinx, 8.5% were white, 8.5% were Asian/Pacific Islander, and 8.5% were multiracial. The average age was 15.8 years old (SD = 1.1). Eighty percent of the participants’ caregivers reported a household income of less than $50,000. The sample analyzed in this study (*n* = 20) included only participants who had complete data on measures of food insecurity and BMI.

*Study 2*: One hundred-nine adults across the Chicagoland area participated in the study. The sample was 70% female, 40% Black, 40% Latinx, 15% white, and 5% other. The average age of the larger sample was 33.12 (*SD* = 11.99). One participant identified as nonbinary. Almost 60% of the participants had a household income of less than $50,000. The sample included in this analysis (*n* = 44) included participants who were under 30 years of age (meeting the criteria for young adults) and had complete data on measures of food insecurity and BMI.

### 2.2. Procedure

*Study 1*: Students and caregivers were recruited through a convenience sampling approach. They were approached at school during times when both caregivers and students were typically present, such as orientations and open houses. Students participated in focus groups at school in which they were administered a questionnaire assessing demographic information and health behaviors. Caregivers were administered a similar questionnaire, but gave more information pertaining to socioeconomic status, food availability and home composition. All participants received a $50 Amazon gift card upon completion of the study.

*Study 2*: Participants were recruited through contact tracers at Brother’s Health Collective, a health clinic in the Bronzeville neighborhood of Chicago, and through flyers posted around the Bronzeville area. After completing the informed consent process, participants completed a Qualtrics survey that asked demographic questions and health-related questions, including food insecurity. They were given a $50 gift card for their participation.

### 2.3. Measures

For the purposes of the current study, we were interested in viewing the geospatial relationship between food insecurity and BMI related to proximity to grocery stores and income levels in Chicagoland zip codes. In both studies, we assessed food insecurity, BMI, and several other sociodemographic variables. However, food insecurity and BMI were assessed differently in each study.

*Study 1*: Food security was measured through a 4-item subset of the home food availability questions from the National Health and Nutrition Examination Survey (NHANES). This measure includes statements such as “I don’t buy fruits because they cost too much” and “At the store where I buy my groceries, the variety of fresh fruits and vegetables is limited” and participants were asked to respond on a 4-point Likert scale the extent to which they agreed or disagreed with those statements. “Strongly agree” and “agree” responses were grouped into one category (recoded as “1”) and responses including “disagree” or “strongly disagree” were grouped into a separate category and recoded as “0.” All of the items answered in the affirmative were summed (i.e., *agree* or *strongly agree*) and scores ranged from 1–4. Participants with a score of 1 had some food insecurity but it was low overall, and 4 indicated higher levels of food insecurity.

Study 1 participants were weighed using a digital scale in a private area in the school wearing no shoes and light clothing. BMI was calculated using the following formula: weight in kilograms divided by height in meters squared.

*Study 2*: Food security was measured with the 10-item USDA food security scale. Examples of questions included “I worried whether my food would run out before I got money to buy more,” and “In the last 12 months, were you ever hungry but didn’t eat because there wasn’t enough money for food?”. The measure was scored by summing all of the items answered in the affirmative (i.e., the number of questions to which participants responded with *often true* or *sometimes true*). Our score categories for this variable map onto the USDA’s guidelines for categorizing food insecurity with this measure. For example, a score of 0 was consistent with being food secure, 1–2 was food insecure without hunger, 3–5 was food insecure with hunger (moderate), and 5–7 was food insecure with hunger (severe).

In Study 2, due to restrictions from the COVID-19 pandemic, participants self-reported their weight on the questionnaire, which was then used to calculate BMI using the same formula as Study 1.

### 2.4. Creation of Maps

**Figure 1:**
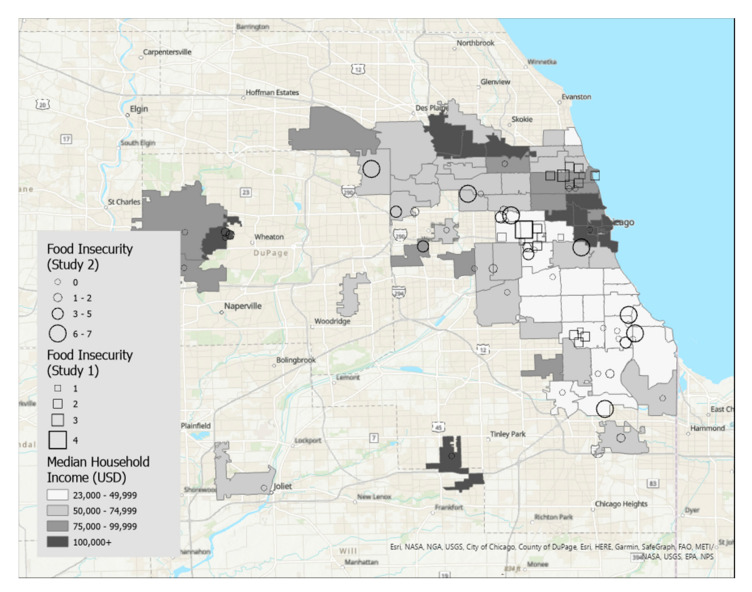
This figure displays food insecurity overlaid on a map of median income. The map shows that participants with the highest food insecurity (larger symbols) tend to live in the zip codes with the lowest median income. In contrast, participants with low food insecurity were dispersed broadly across zip codes, with participants represented in all income categories.

**Figure 2:**
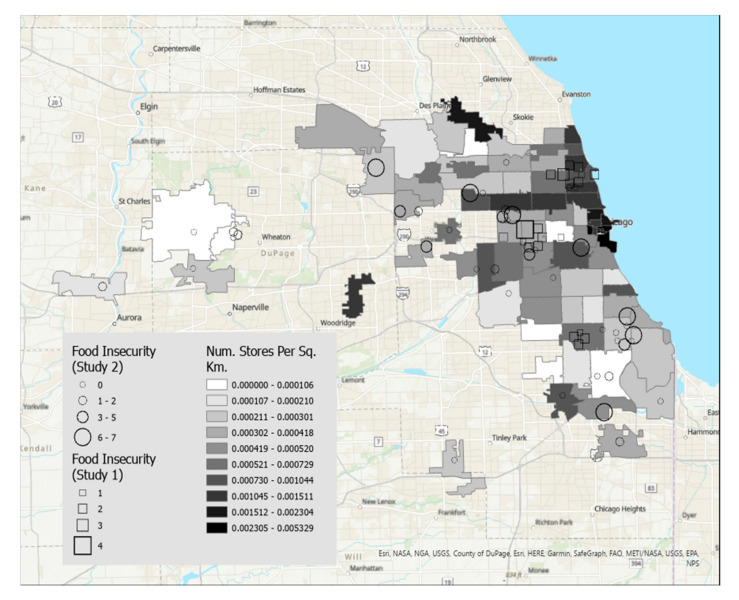
This figure displays food insecurity overlaid on a map of store density (darker shade indicates higher density). There did not appear to be clear a relationship between food insecurity and store density.

**Figure 3:**
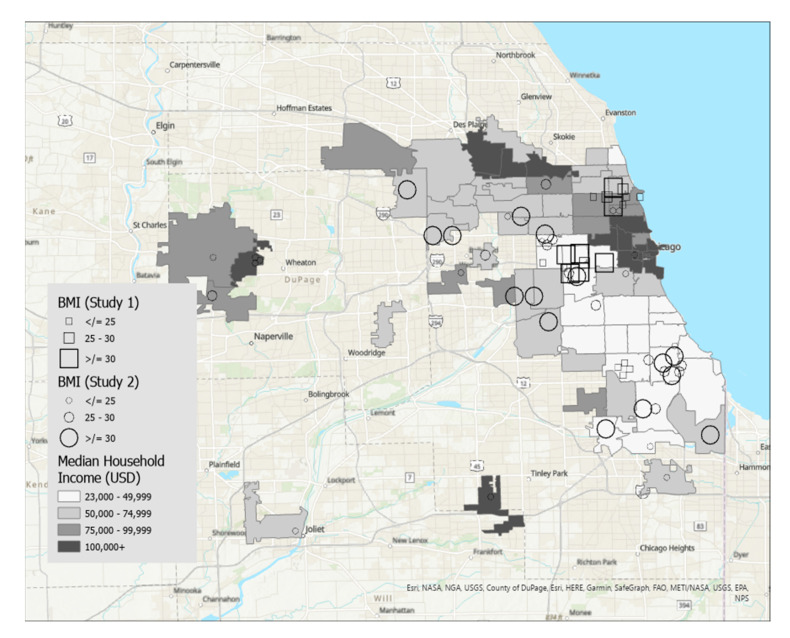
This figure displays BMI overlaid on a map of median income. Participants with the highest BMI tend to live in zip codes with lower median income. Similarly, participants with lower BMIs were dispersed across zip codes with varying levels of median income.

**Figure 4:**
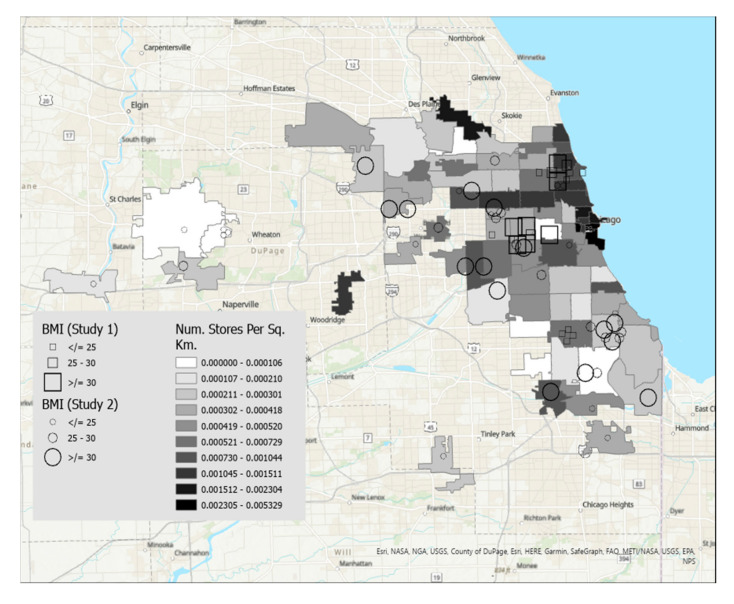
This figure displays BMI overlaid on a map of store density (darker shade indicates higher density). A pattern emerged such that participants with higher BMI tended to live in the south and west sides of Chicago, which have a relatively lower concentration of grocery stores in the city. Participants who lived in suburban zip codes tended to have lower BMI. Thus, city-dwelling participants with fewer grocery stores in their zip codes tended to have high BMIs.

Maps were created using ArcGIS Pro, a desktop geographic information system software [16]. Figure 1 displays food insecurity overlaid on zip codes of the Chicago, IL metropolitan area that were shaded by median household income, and Figure 2 displays food insecurity overlaid on zip codes shaded according to density of grocery stores per square kilometer. Figure 3 displays BMI overlaid on zip codes of the Chicago, IL metropolitan area that were shaded by median household income. Figure 4 displays BMI shaded by grocery stores per square kilometer. Details regarding variable categories and visual representations can be found in each figure’s corresponding legend and figure caption.

Although the Chicago metropolitan area consists of more zip codes than were displayed in the figures, only the suburban zip codes that were represented in our datasets were included in the analyses for ease of interpretation. All zip codes contained in the city of Chicago were included. Geographic boundaries for each zip code were extracted from geospatial data files downloaded from the U.S. Government’s open data repository [17]. Data for each zip code’s median household income and land area were drawn from the U.S. Census Bureau [18]. Grocery store density in each zip code was calculated by dividing the number of grocery stores by the land area in square kilometers. Grocery stores were defined as stores selling primarily a range of food products, including whole fruits and vegetables, and excluded gas stations, convenience stores, or liquor stores. Data on the frequency of grocery stores per zip code were drawn from the City of Chicago’s data portal [19].

Each participant is represented by a single symbol on the maps. Participants from the high school student participants (Study 1, *n* = 20) were represented by squares, and participants from the community sample of young adults (Study 2, *n* = 44) were represented by circles.

### 2.5. Regression Analyses

While the purpose of this paper was to display spatial relationships between food insecurity and BMI and median income and store density, we conducted linear regression and logistic regression analyses, respectively, to determine whether statistically significant relationships exist between these variables. For the logistic regression analysis, we recoded the food insecurity variable into 0 (low food insecurity) and 1 (high food insecurity) based on the median split for each measure.

## 3. Results

### 3.1. Food Insecurity Results

Figure 1 displays food insecurity overlaid on a map of median income. The map shows that participants with the highest food insecurity (larger symbols) tend to live in the zip codes with the lowest median income. In contrast, participants with low food insecurity were dispersed broadly across zip codes, with participants represented in all income categories. Figure 2 displays food insecurity overlaid on a map of store density. There did not appear to be clear a relationship between food insecurity and store density.

Logistic regression reveals that store density (X^2^ (1, *n* = 65) = 1.01, *p* = 0.32) and median income (X^2^ (1, *n* = 65) = 0.037, *p* = 0.85) did not significantly increase the likelihood of having higher levels of food insecurity.

### 3.2. BMI Results

Figure 3 displays BMI overlaid on a map of median income. Participants with the highest BMI tend to live in zip codes with lower median income. Similarly, participants with lower BMIs were dispersed across zip codes with varying levels of median income. Figure 4 displays BMI overlaid on a map of store density. A pattern emerged such that participants with higher BMI tended to live in the south and west sides of Chicago, which have a relatively lower concentration of grocery stores in the city. Participants who lived in suburban zip codes tended to have lower BMI. Thus, city-dwelling participants with fewer grocery stores in their zip codes tended to have high BMIs.

Linear regression analysis revealed that BMI was significantly and positively associated with median income (*r*(63) = −0.353 **, *p* = 0.004) but not with store density (*r*(63) = −0.151, *p* = 0.230).

Of note, we did also map the data for both food insecurity and BMI based on race/ethnicity and sex but did not see any patterns in terms of our variables of interest, so we dropped these demographic variables to facilitate the interpretation of the maps.

## 4. Discussion

Food insecurity occurs when a household lacks consistent access to food and is more prevalent in ethnic and racial minoritized populations. While there has been a proliferation of research linking food insecurity to obesity [1,2,4,5,8,9], these findings are mixed. Given the mixed findings from traditional quantitative approaches, we used GIS approaches to visualize additional geographic factors in Chicago and the surrounding area that may be associated with food insecurity and BMI. GIS analysis revealed that participants with the highest food insecurity tend to live in the zip codes with the lowest median income. This finding is consistent with broader national findings that food insecurity is closely linked to lower income levels [20]. In contrast, participants with low food insecurity were dispersed broadly across zip codes, with participants represented in all income categories. Those with high food insecurity tended to live in areas with lower median income. These findings are interesting and suggests that the relationship between income and high food insecurity may be stronger for those living in poverty whereas the relationship between income and low food insecurity may be less clear. Consistent with previous literature [21,22], there did not appear to be a clear pattern between food insecurity and store density. Some of the smaller shapes, indicating lower levels of food insecurity, do tend to be concentrated on the north side, which has a high concentration of stores, but the patterns are less clear in other areas of the city and suburbs. It may also be that the north side also has a higher level of income, which is really driving the picture of food insecurity above and beyond the concentration of stores. Our study only included grocery stores and excluded other types of stores where individuals living in food deserts may go to shop such as convenience stores and gas stations. While we wanted to limit this analysis to stores where fresh fruits and vegetables are more likely to be sold, there may be a wide range of accessibility to nutritious foods across these convenience stores that is not captured in our data.

Regarding BMI, participants with the highest BMI tend to live in zip codes with lower median incomes, which is consistent with previous literature [23]. Similar to our food insecurity findings, however, participants with lower BMIs were dispersed across zip codes with varying levels of median income. This finding may suggest that the relationship between income and high BMI may be stronger for those living in poverty, whereas the relationship between income and lower BMI may be less clear. Additionally, a pattern emerged such that participants with higher BMI tended to live in the south and west sides of Chicago, which have a relatively lower concentration of grocery stores in the city. Participants who lived in suburban zip codes tended to have lower BMI. Thus, city-dwelling participants with fewer grocery stores in their zip codes tended to have higher BMIs. While we also mapped sex and race/ethnicity on each of these maps, we did not find any clear patterns as these sociodemographic variables related to BMI/food insecurity as they relate to income and store density. This was surprising given previous research; however, while our maps did highlight areas of segregation and economic inequities across the city, there were no visible patterns between race/ethnicity and our variables of interest (BMI/Food insecurity), and income showed more of a clear pattern among our participants across studies.

Regarding inferential statistics findings, BMI was significantly and positively associated with median income but not with store density. Store density and median income did not significantly increase the likelihood of having higher levels of food insecurity. The finding that BMI was significantly associated with income has been demonstrated consistently in previous research. It is difficult to interpret the other findings given the small size of our sample. In other words, it is difficult to determine if these relationships do not exist, or that we were underpowered to find differences. Thus, it is important to replicate these methods with a larger sample of participants.

Our GIS findings may help to inform future interventions and policy approaches to addressing both obesity and food insecurity in areas of higher prevalence. Through our mapping, we can see the health inequities that exist, particularly in the south and west sides. These areas are high poverty areas with known food deserts. Our findings suggest that it may be helpful to develop community-engaged interventions in these geographic regions to help address obesity and food insecurity related inequities. One strength of GIS analysis is that it creates a clear picture of where inequities lie that is easily interpretable for community members. Building relationships with community partners in high-risk regions and utilizing community-engaged methods to develop and implement interventions given our findings may be an important next step to addressing neighborhood-level social determinants of health driving inequities in obesity and food insecurity. Given that we found patterns between food insecurity and BMI on the income maps, it suggests that income-related inequalities have broader health consequences that should be addressed. In Cook County, a guaranteed income pilot was recently launched to determine whether economic and other inequities in Chicago may be mitigated by universal income [24]. Future research should look at how this initiative impacts BMI/food insecurity as well as other economic and social factors. Our findings may also suggest that it may be important to address food and nutrition deserts across large cities to ensure that those residing outside of the most congested and wealthy areas of the city have close access to healthful foods. Infrastructure building may also mitigate the harms of food deserts by facilitating transit to and from stores. Future research may also consider quantitative methods to determine whether links between income food insecurity/BMI may differ for those with high and low levels of each.

Our study should be considered within the context of some important limitations. First, our GIS analysis is limited to one US metropolitan and surrounding area. While it may be generalizable to other similar-sized cities, it may not be generalizable to other smaller-sized cities in other geographic regions of the US. Further, we had a relatively small sample size overall. This limited our ability to conduct regression analyses to determine definitively if there was a statistically significant relationship between BMI and food insecurity and store density/median income. Future research should replicate our methods with larger samples of participants. Additionally, we did not have exact locations of our participants (only zip codes) which limited our ability to conduct a spatial logistic regression. Our sample was also largely comprised of minoritized adolescents and young adults of low-income which is important given the aims of our study but limit the generalizability to other incomes and racial and ethnic groups. Another limitation is that food insecurity was measured using two different measures across studies. While our maps depict the range of scores on each, it is possible that there is some measurement variability across scales. Finally, Study 2 calculated BMI from self-reported height and weight rather than objective measures.

Despite its limitations, our study contributes to the literature by using GIS methods rather than typical quantitative measurement strategies so that geographic variations in patterns of these relations are not overlooked. As we see with our results, there are wide ranges of findings across relatively small geographic regions highlighting areas of highest inequity. Average scores mask these geographic differences making it difficult to develop tailored intervention and policy approaches to addressing inequities in areas of the greatest need. We also shed some light on the complicated relationship between food insecurity and BMI by looking at how these vary by income and grocery store density.

## 5. Conclusions

Our study suggests that there are geographic inequities across neighborhoods that exist, pointing to a need for greater nuance in the development of intervention and policy approaches to address obesity and food insecurity. Future research may replicate this approach in other cities to see if findings are consistent and may call for more federal level policy approaches to address disparities across neighborhoods in cities who have high levels of health inequity.

## Data Availability

Data supporting reported results can be found at the following publicly available archive: https://zenodo.org/record/7629854 (accessed on 14 February 2023).
